# Multiple sclerosis health-related quality of life utility values from the UK MS register

**DOI:** 10.1177/20552173231178441

**Published:** 2023-06-02

**Authors:** A Heather, E Goodwin, C Green, N Morrish, OC Ukoumunne, RM Middleton, A Hawton

**Affiliations:** PenCHORD (The Peninsula Collaboration for Health Operational Research and Data Science), Department of Health and Community Sciences, 3286 University of Exeter, Exeter, UK; Health Economics Group, Department of Health and Community Sciences, 3286 University of Exeter, Exeter, UK; Health Economics Group, Department of Health and Community Sciences, 3286 University of Exeter, Exeter, UK; Department for Neurobiology, Care Sciences and Society, Center for Alzheimer Research, 27106 Karolinska Institutet, Solna, Sweden; Biogen UK & Ireland, Berkshire, UK; Health Economics Group, Department of Health and Community Sciences, 3286 University of Exeter, Exeter, UK; NIHR Applied Research Collaboration South West Peninsula, Department of Health and Community Sciences, 3286 University of Exeter, Exeter, UK; Population Data Science, 7759 Swansea University, UK; Health Economics Group, Department of Health and Community Sciences, 3286 University of Exeter, Exeter, UK; NIHR Applied Research Collaboration South West Peninsula, Department of Health and Community Sciences, 3286 University of Exeter, Exeter, UK

**Keywords:** Multiple sclerosis, cost-benefit analysis, patient-reported outcome measures, quality of life, technology assessment, biomedical, United Kingdom

## Abstract

**Background:**

New interventions for multiple sclerosis (MS) commonly require a demonstration of cost-effectiveness using health-related quality of life (HRQoL) utility values. The EQ-5D is the utility measure approved for use in the UK NHS funding decision-making. There are also MS-specific utility measures – e.g., MS Impact Scale Eight Dimensions (MSIS-8D) and MSIS-8D-Patient (MSIS-8D-P).

**Objectives:**

Provide EQ-5D, MSIS-8D and MSIS-8D-P utility values from a large UK MS cohort and investigate their association with demographic/clinical characteristics.

**Methods:**

UK MS Register data from 14,385 respondents (2011 to 2019) were analysed descriptively and using multivariable linear regression, with self-report Expanded Disability Status Scale (EDSS) scores.

**Results:**

The EQ-5D and MSIS-8D were both sensitive to differences in demographic/clinical characteristics. An inconsistency found in previous studies whereby mean EQ-5D values were higher for an EDSS score of 4 rather than 3 was not observed. Similar utility values were observed between MS types at each EDSS score. Regression showed EDSS score and age were associated with utility values from all three measures.

**Conclusions:**

This study provides generic and MS-specific utility values for a large UK MS sample, with the potential for use in cost-effectiveness analyses of treatments for MS.

## Introduction

New interventions for multiple sclerosis (MS) commonly require a demonstration of efficacy, safety and cost-effectiveness. Assessment of cost-effectiveness often involves weighing the relative costs of an intervention against the relative impact on health-related quality of life (HRQoL). In cost-effectiveness analyses, utility values are used to quantify HRQoL. They typically range from one to zero, where one represents perfect health and zero is considered equivalent to being dead. Negative utility values are also possible, representing health states considered less preferable than death. Utility values are commonly obtained using preference-based measures (PBMs). These comprise two components: (i) a descriptive system which captures an individual's health on a number of dimensions (e.g., mobility, pain), each with a number of severity levels, with each combination of dimensions/levels constituting a health state and; (ii) a tariff of utility values for each health state.^1^ The EQ-5D^2^ is the most widely used PBM and is recommended by the National Institute for Health and Care Excellence (NICE) for use in considering which interventions should be made available on the UK NHS.^3^ Utility values are used in cost-effectiveness analyses to calculate quality-adjusted life-years (QALYs) which quantify the impact of an intervention on quality and duration of life.^1^ When utility values are not available for the participants of a clinical trial, values reported in the literature can be used.^3^

There are inconsistencies in the EQ-5D values reported for people with MS. There is inconsistency in the literature with regards to whether primary progressive MS (PPMS) or secondary progressive MS (SPMS) have the lowest EQ-5D values.^4–7^ There are also inconsistencies in the EQ-5D values observed between different disease severities. MS severity is typically measured using the Expanded Disability Status Scale (EDSS). EDSS scores can range from 0 to 10 in 0.5 increments, where 0 represents a normal neurological examination and 10 represents death due to MS.^8^ EDSS score is often based on a clinician assessment, but self-report tools are also available, as is the case for the dataset in the present study. It is anticipated that EQ-5D values would decrease as EDSS scores increase, but multiple studies have found higher mean EQ-5D values for an EDSS score of 4 than for a score of 3.^4,6,9^ The face validity of these values and the discriminative validity of the EQ-5D for MS has been questioned, leading to concerns about the relevance and responsiveness of the EQ-5D to MS. The EQ-5D has also been found to lack content validity in MS as it does not capture fatigue and cognition.^10^

Condition-specific PBMs (CS-PBMs) offer an alternative to generic PBMs like the EQ-5D. They capture aspects of HRQoL that are specifically relevant to people with a particular condition, meaning that they may be more sensitive to changes in the HRQoL of people with that condition.^11^ CS-PBMs may be approved by NICE for use in cost-effectiveness analyses in situations where a generic PBM is considered inappropriate.^3^ Reported MS-specific PBMs include the MS Impact Scale Eight Dimensions (MSIS-8D),^12^ the MS Impact Scale PBM (MSIS-PBM),^11^ the Preference-Based MS Index (PBMSI)^13–15^ and the Neuro-QoL Utility Scoring System (NQU).^16^ These measures have been developed using items from established non-preference-based outcome measures, so can be used retrospectively to obtain utility values.

NICE requires that tariffs of utility values are based on the preferences of the general population,^3^ in line with the argument that the values used should reflect the preferences of the broad population of taxpayers. However, there are also arguments for the use of preferences from people with MS, such as that they will be informed by lived experiences of the condition and that it is people with MS who will be most impacted by the outcomes of appraisals using these values.^17–19^ A tariff of utility values based on preferences elicited from people with MS is available for the MSIS-8D – the MSIS-8D-Patient Version (MSIS-8D-P).^20^

The current research aims to: (i) provide utility values from the EQ-5D, MSIS-8D and MSIS-8D-P based on a large, recent UK cohort of people with MS using data from the UK MS Register and (ii) investigate the association between these utility values and demographic and clinical characteristics.

## Methods

The UK MS Register is a prospective cohort study launched to online participants in May 2011. Ethical approval was obtained from the South West Central Bristol Research Ethics Council (initially as 11/SW/0160, then 16/SW/0194, now 21/SW/0085). The study conducts biannual surveys of people with neurologist-confirmed MS aged 18 or over, resident in the UK via a web portal. In addition to demographic and clinical data, the UK MS Register collects responses to the EQ-5D-3L and the MSIS-8D (Table 1). Disease severity is reported using the self-report web-EDSS, as described by Leddy et al. (2013).^21^ The UK MS Register population was found to be representative of the wider UK population of people with MS.^22^ The present study utilises responses obtained up to April 2019. Answers to the EDSS, MSIS-8D, MSIS-8D-P and EQ-5D were contemporaneous.

**Table 1.** Description of the EQ-5D-3L, MSIS-8D and MSIS-8D-P.

Measure	Dimensions	Number of levels that each dimension is scored on	Source of the tariff of utility values	Range of utility values
EQ-5D-3L^2^	Five dimensions: • Mobility • Self-care • Usual activities • Pain/Discomfort Anxiety/Depression	Three	Using the time-trade-off technique in a sample of the UK general population^2^	−0.594 to 1
MSIS-8D^12^	Eight dimensions: • General physical function • Mobility • Employment • Social function • Fatigue • Cognition • Depression • General emotional well-being	Four	Using the time-trade-off technique in a sample of the UK general population^12^ and people with MS (MSIS-8D-P)^20^	MSIS-8D: 0.079 to 0.882 MSIS-8D-P: 0.138 to 0.893

MS, multiple sclerosis; MSIS-8D, Multiple Sclerosis Impact Scale Eight Dimensions; MSIS-8D-P, Multiple Sclerosis Impact Scale Eight Dimensions Patient Version.

Data preparation and analysis were undertaken using Stata Version 15.1. Descriptive statistics including mean and standard deviation (SD) were used to summarise sample characteristics and utility values observed by demographic and clinical characteristics (for cells of *n* > 30). The proportion of people with each type of MS was reported by gender and current age. To investigate the strength of the relationship between each of the demographic and clinical variables and the utility values, whilst controlling for the impact of the other demographic and clinical characteristics, a multivariable linear regression analysis was conducted. This was based on a sub-group of data from individuals at the first timepoint that they provided complete data for the EQ-5D, MSIS-8D, MSIS-8D-P and the relevant clinical and demographic characteristics. Robust regression was used as the residuals did not meet the assumptions of normality and homoscedasticity. Age and time since diagnosis were centred.

## Results

The sample consisted of 14,385 participants who provided at least one response to at least one PBM. The sample demographic and clinical characteristics are summarised in Table 2. The mean age was 55.3 years (SD 11.4) and 72.8% of participants were female. The mean EDSS score was 5.1 (SD 2.0), based on an average of 1.8 responses per person from 3937 participants. The mean EQ-5D, MSIS-8D and MSIS-8D-P values were 0.562 (SD 0.308), 0.603 (SD 0.18) and 0.643 (SD 0.173) respectively, based on an average of 4.4 responses per person. The full range of possible values was observed for each measure. Only results from the EQ-5D and MSIS-8D are described below since differences in the MSIS-8D and MSIS-8D-P between different demographic and clinical characteristics (Tables 3–4) and regression results for the two measures (Table 5) were very similar.

**Table 2.** Demographic and clinical characteristics of the study sample (*N*, number of responses; *n*, number of participants).

Characteristics	Whole, prospective cohort^a,b^	Cross-sectional sub-group for multivariable regression^b,c^
*N*	14,385	2968
Gender, % (*n*)		
Female	72.8 (9780)	70.8 (2102)
Male	27.2 (3655)	29.2 (866)
Current age, years, mean (SD) (*N*, *n*)	55.3 (11.4) (64,671, 13,340)	55.1 (11.7) (2968, 2968)
Current age, years, % (*N*, *n*)		
<25	0.2 (138, 59)	0.3 (9, 9)
25 to 29	1.1 (693, 276)	1.4 (42, 42)
30 to 34	2.7 (1758, 645)	3.5 (105, 105)
35 to 39	5.5 (3538, 1206)	5.5 (162, 162)
40 to 44	8.5 (5503, 1725)	8.0 (237, 237)
45 to 49	12 (7763, 2446)	12.2 (362, 362)
50 to 54	15.3 (9926, 2900)	14.5 (429, 429)
55 to 59	16.9 (10,916, 2848)	17.3 (514, 514)
60 to 64	15.3 (9910, 2456)	15.3 (453, 453)
65 to 69	11.8 (7623, 1860)	11.2 (331, 331)
70 to 74	7.3 (4746, 1198)	7.4 (220, 220)
75 to 79	2.5 (1629, 443)	2.5 (73, 73)
80 ≤	0.8 (528, 149)	1.0 (31, 31)
Age at diagnosis, years, mean (SD) (*n*)	39.2 (10.4) (12,244)	40.9 (10.6) (2968)
Current MS, % (*N*, *n*)		
RRMS	55.0 (30,262, 6037)	56.7 (1683, 1683)
SPMS	26.6 (14,634, 2521)	26.1 (776, 776)
PPMS	18.4 (10,141, 1588)	17.1 (509, 509)
MS at diagnosis, % (*n*)		
RRMS	77.3 (7211)	75.6 (2077)
SPMS	8.0 (742)	8.2 (225)
PPMS	14.7 (1372)	16.2 (446)
Time since diagnosis, years, mean (SD) (*N*, *n*)	14.8 (9.3) (61,764, 12,155)	14.2 (9.4) (2968, 2968)
Time since diagnosis, years, % (*N*, *n*)		
<2	0.7 (437, 261)	2.9 (87, 87)
2 to 4	9.2 (5667, 1893)	11.1 (328, 328)
5 to 9	25.3 (15,620, 4128)	23.3 (692, 692)
10 to 14	21.5 (13,268, 3639)	21.1 (625, 625)
15 to 19	16.4 (10,114, 2685)	15.3 (455, 455)
20 to 24	11.4 (7028, 1834)	10.9 (324, 324)
25 to 29	7.8 (4794, 1199)	7.9 (233, 233)
30 to 34	4.2 (2601, 649)	4.1 (122, 122)
35 to 39	2.0 (1252, 344)	2.2 (66, 66)
40 to 44	0.9 (586, 164)	0.7 (21, 21)
45 ≤	0.6 (397, 88)	0.5 (15, 15)
EDSS, mean (SD) (*N*, *n*)	5.1 (2.0) (7283, 3937)	5.1 (2.1) (2968, 2968)
EDSS, grouped, % (*N*, *n*)		
0	3.3 (237, 180)	3.4^d^ (—^e^)
1 or 1.5	0.8 (59, 46)	0.9^d^ (—^e^)
2 or 2.5	11.5 (835, 626)	11.5^d^ (—^e^)
3 or 3.5	11.1 (810, 594)	11.8^d^ (—^e^)
4 or 4.5	12.6 (920, 688)	12.3^d^ (—^e^)
5 or 5.5	6.2 (448, 364)	6.2^d^ (—^e^)
6 or 6.5	35.0 (2552, 1491)	33.6^d^ (—^e^)
7 or 7.5	16.4 (1195, 730)	17.2^d^ (—^e^)
8 or 8.5	3.1 (226, 148)	3.1^d^ (—^e^)
9 or 9.5	—^f^	—^f^
EQ-5D, mean (SD) [min to max]^g^ (*N*, *n*)	0.562 (0.308) [−0.594 to 1] (*N* = 61,126, *n *= 13,830)	0.585 (0.305) [−0.594, 1] (*N* = 2968, *n* = 2968)
MSIS-8D, mean (SD) [min to max]^h^ (*N*, *n*)	0.603 (0.180) [0.079 to 0.882] (*N* = 61,557, *n* = 13,888)	0.616 (0.181) [0.079, 0.882] (*N* = 2968, *n* = 2968)
MSIS-8D-P, mean (SD) [min to max]^i^ (*N*, *n*)	0.643 (0.173) [0.138 to 0.893] (*N* = 61,557, *n* = 13,888)	0.657 (0.174) [0.138, 0.893] (*N* = 2968, *n* = 2968)

EDSS, Expanded Disability Status Scale; MS, multiple sclerosis; *N*, number of responses; *n*, number of participants; PPMS, primary progressive multiple sclerosis; RRMS, relapsing-remitting multiple sclerosis; SD, standard deviation; SPMS, secondary progressive multiple sclerosis.

^a^ Proportions of each characteristic may not sum to total cohort size (*n* = 14,385) due to missing data on demographic and clinical characteristics for some individuals.

^b^ Percentages may not sum to 100.0 due to rounding.

^c^ Cross-sectional sub-group of individuals with complete data for the EQ-5D, MSIS-8D, MSIS-8D-P and the relevant clinical and demographic characteristics.

^d^ Percentages calculated with the exclusion of EDSS 9 or 9.5, so that the exact number of individuals with an EDSS score of 9 or 9.5 cannot be deduced.

^e^ Not reported so that exact number of individuals with an EDSS score of 9 or 9.5 cannot be deduced, as there are fewer than 5.

^f^ Not reported as observations available from fewer than five participants.

^g^ Possible scores range from −0.594 to 1.

^h^ Possible scores range from 0.079 to 0.882.

^i^ Possible scores range from 0.138 to 0.893.

**Table 3.** Mean utility values by demographic and clinical characteristics.

	Mean (SD) [Min to Max] (*N*, *n*)		
	EQ-5D	MSIS-8D	MSIS-8D-P
EDSS score			
0	0.906 (0.152) [0.157 to 1] (*N* = 222, *n* = 171)	0.806 (0.091) [0.382 to 0.882] (*N* = 211, *n* = 163)	0.831 (0.08) [0.441 to 0.893] (*N* = 211, *n* = 163)
1 or 1.5	0.904 (0.11) [0.414 to 1] (*N* = 52, *n* = 41)	0.802 (0.079) [0.565 to 0.882] (*N* = 49, *n* = 39)	0.83 (0.069) [0.611 to 0.893] (*N *= 49, *n* = 39)
2 or 2.5	0.849 (0.167) [0.024 to 1] (*N* = 768, *n* = 583)	0.774 (0.104) [0.243 to 0.882] (*N* = 787, *n* = 597)	0.806 (0.093) [0.278 to 0.893] (*N* = 787, *n* = 597)
3 or 3.5	0.82 (0.152) [0.151 to 1] (*N* = 749, *n* = 559)	0.753 (0.102) [0.167 to 0.882] (*N* = 754, *n *= 556)	0.787 (0.093) [0.238 to 0.893] (*N* = 754, *n* = 556)
4 or 4.5	0.688 (0.205) [−0.19 to 1] (*N* = 824, *n* = 621)	0.675 (0.132) [0.079 to 0.882] (*N* = 839, *n* = 643)	0.716 (0.126) [0.138 to 0.893] (*N* = 839, *n* = 643)
5 or 5.5	0.575 (0.246) [−0.181 to 1] (*N* = 404, *n* = 335)	0.61 (0.163) [0.079 to 0.882] (*N* = 405, *n* = 328)	0.652 (0.157) [0.138 to 0.893] (*N* = 405, *n* = 328)
6 or 6.5	0.503 (0.255) [−0.319 to 1] (*N* = 2330, *n* = 1393)	0.564 (0.167) [0.079 to 0.882] (*N* = 2345, *n* = 1408)	0.608 (0.162) [0.138 to 0.893] (*N* = 2345, *n* = 1408)
7 or 7.5	0.35 (0.307) [−0.594 to 1] (*N* = 1094, *n* = 683)	0.486 (0.174) [0.079 to 0.882] (*N* = 1097, *n* = 684)	0.533 (0.17) [0.138 to 0.893] (*N* = 1097, *n* = 684)
8 or 8.5	0.16 (0.264) [−0.358 to 0.71] (*N* = 205, *n* = 141)	0.452 (0.173) [0.079 to 0.845] (*N* = 209, *n* = 141)	0.5 (0.169) [0.138 to 0.865] (*N* = 209, *n* = 141)
9 or 9.5	—^a^	—^a^	—^a^
Current MS			
RRMS	0.652 (0.28) [−0.594 to 1] (*N* = 27,714, *n* = 5949)	0.646 (0.173) [0.079 to 0.882] (*N* = 27,934, *n* = 5914)	0.684 (0.165) [0.138 to 0.893] (*N* = 27,934, *n* = 5914)
SPMS	0.43 (0.301) [−0.594 to 1] (*N* = 13,450, *n* = 2473)	0.535 (0.17) [0.079 to 0.882] (*N* = 13,407, *n* = 2450)	0.58 (0.165) [0.138 to 0.893] (*N* = 13,407, *n* = 2450)
PPMS	0.489 (0.295) [−0.594 to 1] (*N* = 9341, *n* = 1567)	0.565 (0.173) [0.079 to 0.882] (*N* = 9371, *n* = 1562)	0.61 (0.167) [0.138 to 0.893] (*N* = 9371, *n* = 1562)
Age, years			
< 25	0.598 (0.347) [−0.331 to 1] (*N* = 117, *n* = 57)	0.63 (0.177) [0.119 to 0.882] (*N* = 120, *n* = 57)	0.67 (0.171) [0.166 to 0.893] (*N* = 120, *n* = 57)
25 to 29	0.657 (0.299) [−0.239 to 1] (*N* = 626, *n* = 269)	0.637 (0.181) [0.079 to 0.882] (*N* = 632, *n* = 263)	0.674 (0.171) [0.138 to 0.893] (*N* = 632, *n* = 263)
30 to 34	0.655 (0.31) [−0.239 to 1] (*N* = 1570, *n* = 629)	0.639 (0.189) [0.079 to 0.882] (*N* = 1625, *n* = 622)	0.677 (0.18) [0.138 to 0.893] (*N* = 1625, *n* = 622)
35 to 39	0.668 (0.299) [−0.594 to 1] (*N* = 3228, *n* = 1165)	0.641 (0.187) [0.079 to 0.882] (*N* = 3278, *n* = 1171)	0.678 (0.176) [0.138 to 0.893] (*N* = 3278, *n* = 1171)
40 to 44	0.643 (0.302) [−0.594 to 1] (*N* = 4992, *n* = 1656)	0.64 (0.184) [0.079 to 0.882] (*N* = 5093, *n* = 1679)	0.677 (0.175) [0.138 to 0.893] (*N* = 5093, *n* = 1679)
45 to 49	0.59 (0.307) [−0.594 to 1] (*N* = 7057, *n* = 2364)	0.612 (0.182) [0.079 to 0.882] (*N* = 7158, *n* = 2365)	0.652 (0.174) [0.138 to 0.893] (*N* = 7158, *n* = 2365)
50 to 54	0.567 (0.304) [−0.594 to 1] (*N* = 9123, *n* = 2825)	0.599 (0.183) [0.079 to 0.882] (*N* = 9213, *n* = 2824)	0.64 (0.176) [0.138 to 0.893] (*N *= 9213, *n* = 2824)
55 to 59	0.535 (0.306) [−0.594 to 1] (*N* = 10,058, *n* = 2763)	0.584 (0.183) [0.079 to 0.882] (*N* = 10,030, *n* = 2767)	0.626 (0.176) [0.138 to 0.893] (*N* = 10,030, *n* = 2767)
60 to 64	0.513 (0.311) [−0.594 to 1] (*N* = 9166, *n* = 2397)	0.58 (0.178) [0.079 to 0.882] (*N* = 9152, *n* = 2386)	0.622 (0.171) [0.138 to 0.893] (*N* = 9152, *n* = 2386)
65 to 69	0.539 (0.293) [−0.594 to 1] (*N* = 7039, *n* = 1800)	0.599 (0.17) [0.079 to 0.882] (*N* = 7019, *n* = 1796)	0.642 (0.164) [0.138 to 0.893] (*N* = 7019, *n* = 1796)
70 to 74	0.545 (0.291) [−0.594 to 1] (*N* = 4372, *n* = 1154)	0.606 (0.166) [0.079 to 0.882] (*N* = 4359, *n* = 1167)	0.649 (0.16) [0.138 to 0.893] (*N* = 4359, *n* = 1167)
75 to 79	0.564 (0.285) [−0.594 to 1] (*N* = 1463, *n* = 425)	0.632 (0.152) [0.079 to 0.882] (*N* = 1469, *n* = 425)	0.673 (0.146) [0.138 to 0.893] (*N* = 1469, *n* = 425)
80 ≤	0.553 (0.251) [−0.429 to 1] (*N* = 486, *n* = 144)	0.629 (0.134) [0.079 to 0.882] (*N* = 475, *n* = 144)	0.669 (0.13) [0.138 to 0.893] (*N* = 475, *n* = 144)
Time since diagnosis, years			
< 2	0.677 (0.283) [−0.239 to 1] (*N* = 389, *n* = 250)	0.664 (0.171) [0.079 to 0.882] (*N* = 394, *n* = 253)	0.701 (0.166) [0.138 to 0.893] (*N* = 394, *n* = 253)
2 to 4	0.657 (0.278) [−0.594 to 1] (*N* = 5158, *n* = 1840)	0.649 (0.168) [0.079 to 0.882] (*N* = 5198, *n* = 1838)	0.688 (0.16) [0.138 to 0.893] (*N* = 5198, *n* = 1838)
5 to 9	0.611 (0.3) [−0.594 to 1] (*N* = 14,419, *n* = 4027)	0.62 (0.185) [0.079 to 0.882] (*N* = 14,477, *n* = 4027)	0.66 (0.177) [0.138 to 0.893] (*N* = 14,477, *n* = 4027)
10 to 14	0.571 (0.298) [−0.594 to 1] (*N* = 12,207, *n* = 3541)	0.6 (0.182) [0.079 to 0.882] (*N* = 12,329, *n* = 3546)	0.641 (0.175) [0.138 to 0.893] (*N* = 12,329, *n* = 3546)
15 to 19	0.535 (0.303) [−0.594 to 1] (*N* = 9299, *n* = 2609)	0.59 (0.177) [0.079 to 0.882] (*N* = 9312, *n* = 2613)	0.632 (0.17) [0.138 to 0.893] (*N* = 9312, *n* = 2613)
20 to 24	0.512 (0.309) [−0.594 to 1] (*N* = 6454, *n* = 1782)	0.583 (0.172) [0.079 to 0.882] (*N *= 6475, *n* = 1782)	0.625 (0.166) [0.138 to 0.893] (*N* = 6475, *n *= 1782)
25 to 29	0.509 (0.318) [−0.594 to 1] (*N* = 4370, *n* = 1158)	0.585 (0.176) [0.079 to 0.882] (*N *= 4388, n = 1164)	0.627 (0.169) [0.138 to 0.893] (*N* = 4388, *n* = 1164)
30 to 34	0.512 (0.321) [−0.594 to 1] (*N* = 2395, *n* = 623)	0.592 (0.177) [0.079 to 0.882] (*N* = 2381, *n* = 628)	0.633 (0.17) [0.138 to 0.893] (*N* = 2381, *n* = 628)
35 to 39	0.525 (0.302) [−0.594 to 1] (*N* = 1139, *n* = 330)	0.6 (0.177) [0.079 to 0.882] (*N* = 1123, *n* = 333)	0.642 (0.171) [0.138 to 0.893] (*N* = 1123, *n* = 333)
40 to 44	0.546 (0.292) [−0.484 to 1] (*N* = 526, *n* = 161)	0.598 (0.168) [0.079 to 0.882] (*N* = 547, *n* = 159)	0.64 (0.164) [0.138 to 0.893] (*N* = 547, *n* = 159)
45 ≤	0.573 (0.28) [−0.331 to 1] (*N* = 367, *n* = 84)	0.635 (0.151) [0.079 to 0.882] (*N* = 366, *n* = 86)	0.678 (0.145) [0.138 to 0.893] (*N* = 366, *n* = 86)
Gender			
Male	0.547 (0.308) [−0.594 to 1] (*N* = 16,986, *n* = 3570)	0.596 (0.178) [0.079 to 0.882] (*N* = 17,103, *n* = 3568)	0.636 (0.17) [0.138 to 0.893] (*N* = 17,103, *n* = 3568)
Female	0.571 (0.308) [−0.594 to 1] (*N* = 42,570, *n* = 9554)	0.607 (0.181) [0.079 to 0.882] (*N* = 42,768, *n* = 9501)	0.647 (0.173) [0.138 to 0.893] (*N* = 42,768, *n* = 9501)

EDSS, Expanded Disability Status Scale; Max, maximum; Min, minimum; MS, multiple sclerosis; MSIS-8D, Multiple Sclerosis Impact Scale Eight Dimensions; MSIS-8D-P, Multiple Sclerosis Impact Scale Eight Dimensions Patient Version; N, number of responses; n, number of participants; PPMS, primary progressive multiple sclerosis. RRMS, relapsing-remitting multiple sclerosis; SD, standard deviation; SPMS, secondary progressive multiple sclerosis.

^a^ Not reported as observations available from fewer than 30 participants.

**Table 4.** Mean utility values by EDSS score and MS type.

	Mean (SD) [Min to Max] (*N*, *n*)		
	RRMS	SPMS	PPMS
**EQ-5D** EDSS score			
0	0.907 (0.144) [0.26 to 1] (*N* = 187, *n* = 143)	—^a^	—^a^
1 or 1.5	0.899 (0.113) [0.414 to 1] (*N* = 45, *n* = 35)	—^a^	—^a^
2 or 2.5	0.845 (0.169) [0.024 to 1] (*N* = 558, *n* = 431)	—^a^	—^a^
3 or 3.5	0.815 (0.156) [0.151 to 1] (*N* = 595, *n* = 442)	—^a^	—^a^
4 or 4.5	0.684 (0.216) [−0.19 to 1] (*N* = 570, *n* = 424)	0.684 (0.191) [0.088 to 1] (*N* = 59, *n* = 49)	0.69 (0.167) [−0.112 to 1] (*N* = 96, *n* = 69)
5 or 5.5	0.555 (0.26) [−0.181 to 1] (*N* = 242, *n* = 200)	0.592 (0.228) [−0.016 to 0.883] (*N* = 45, *n* = 36)	0.657 (0.197) [−0.181 to 1] (*N* = 59, *n* = 51)
6 or 6.5	0.498 (0.263) [−0.239 to 1] (*N* = 851, *n* = 511)	0.482 (0.251) [−0.291 to 0.883] (*N* = 769, *n* = 445)	0.55 (0.225) [−0.239 to 1] (*N* = 468, *n* = 280)
7 or 7.5	0.382 (0.299) [−0.349 to 0.85] (*N* = 156, *n* = 105)	0.337 (0.312) [−0.594 to 1] (*N* = 549, *n* = 338)	0.363 (0.307) [−0.594 to 0.85] (*N* = 321, *n* = 192)
8 or 8.5	—^a^	0.139 (0.255) [−0.349 to 0.71] (*N* = 128, *n* = 85)	0.253 (0.286) [−0.349 to 0.71] (N = 45, n = 30)
9 or 9.5	—^a^	—^a^	—^a^
**MSIS-8D** EDSS score			
0	0.808 (0.082) [0.477 to 0.882] (*N* = 178, *n* = 137)	—^a^	—^a^
1 or 1.5	0.805 (0.078) [0.565 to 0.882] (*N* = 43, *n* = 34)	—^a^	—^a^
2 or 2.5	0.767 (0.108) [0.243 to 0.882] (*N* = 576, *n* = 445)	—^a^	—^a^
3 or 3.5	0.751 (0.107) [0.167 to 0.882] (*N* = 600, *n* = 437)	—^a^	—^a^
4 or 4.5	0.671 (0.136) [0.079 to 0.882] (*N* = 577, *n* = 436)	0.674 (0.138) [0.263 to 0.829] (*N* = 57, *n* = 45)	0.68 (0.116) [0.131 to 0.86] (*N* = 101, *n* = 74)
5 or 5.5	0.59 (0.172) [0.079 to 0.882] (*N* = 246, *n* = 198)	0.639 (0.132) [0.276 to 0.882] (*N* = 53, *n* = 42)	0.669 (0.129) [0.225 to 0.882] (*N* = 57, *n* = 49)
6 or 6.5	0.564 (0.172) [0.079 to 0.882] (*N* = 844, *n* = 517)	0.551 (0.158) [0.079 to 0.882] (*N* = 774, *n* = 445)	0.578 (0.164) [0.079 to 0.882] (*N* = 483, *n* = 286)
7 or 7.5	0.465 (0.181) [0.079 to 0.882] (*N* = 154, *n* = 103)	0.484 (0.177) [0.079 to 0.846] (*N* = 542, *n* = 336)	0.501 (0.166) [0.079 to 0.86] (*N* = 332, *n* = 198)
8 or 8.5	—^a^	0.45 (0.179) [0.079 to 0.845] (*N* = 129, *n* = 83)	0.459 (0.146) [0.079 to 0.773] (*N* = 48, *n* = 33)
9 or 9.5	—^a^	—^a^	—^a^
**MSIS-8D-P** EDSS score			
0	0.833 (0.071) [0.476 to 0.893] (*N* = 178, *n* = 137)	—^a^	—^a^
1 or 1.5	0.831 (0.068) [0.611 to 0.893] (*N* = 43, *n* = 34)	—^a^	—^a^
2 or 2.5	0.799 (0.097) [0.278 to 0.893] (*N* = 576, *n* = 445)	—^a^	—^a^
3 or 3.5	0.785 (0.098) [0.238 to 0.893] (*N* = 600, *n* = 437)	—^a^	—^a^
4 or 4.5	0.712 (0.129) [0.138 to 0.893] (*N* = 577, *n* = 436)	0.714 (0.134) [0.312 to 0.846] (*N* = 57, *n* = 45)	0.726 (0.112) [0.174 to 0.89] (*N* = 101, *n* = 74)
5 or 5.5	0.634 (0.166) [0.138 to 0.893] (*N* = 246, *n* = 198)	0.677 (0.128) [0.34 to 0.893] (*N* = 53, *n* = 42)	0.712 (0.127) [0.302 to 0.893] (*N* = 57, *n* = 49)
6 or 6.5	0.608 (0.167) [0.138 to 0.893] (*N* = 844, *n* = 517)	0.596 (0.155) [0.138 to 0.893] (*N* = 774, *n* = 445)	0.624 (0.158) [0.138 to 0.893] (*N* = 483, *n* = 286)
7 or 7.5	0.513 (0.178) [0.138 to 0.893] (*N* = 154, *n* = 103)	0.533 (0.173) [0.138 to 0.863] (*N* = 542, *n* = 336)	0.546 (0.162) [0.138 to 0.893] (*N* = 332, *n* = 198)
8 or 8.5	—^a^	0.498 (0.174) [0.138 to 0.865] (*N* = 129, *n* = 83)	0.507 (0.145) [0.138 to 0.812] (*N* = 48, *n* = 33)
9 or 9.5	—^a^	— ^a^	— ^a^

EDSS, Expanded Disability Status Scale; Max, maximum; Min, minimum; MS, multiple sclerosis; MSIS-8D, Multiple Sclerosis Impact Scale Eight Dimensions; MSIS-8D-P, Multiple Sclerosis Impact Scale Eight Dimensions Patient Version; *N*, number of responses; *n*, number of participants; PPMS, primary progressive multiple sclerosis. RRMS, relapsing-remitting multiple sclerosis; SD, standard deviation; SPMS, secondary progressive multiple sclerosis.

^a^ Not reported as observations available from fewer than 30 participants.

**Table 5.** Results from robust multivariable linear regression for EQ-5D, MSIS-8D and MSIS-8D-P values.

PBM	Predictor	Coefficient	95% CI	P-Value	Beta
EQ-5D	Gender – male	—^a^	—^a^	—^a^	—^a^
	Gender – female	0.002	(−0.018, 0.021)	0.872	0.002
	Current age	0.002	(0.001, 0.003)	0.000	0.088
	Time since diagnosis	0.001	(0.000, 0.002)	0.132	0.027
	Current MS – RRMS	—^a^	—^a^	—^a^	—^a^
	Current MS – PPMS	0.013	(−0.015, 0.041)	0.368	0.016
	Current MS – SPMS	−0.031	(−0.058, −0.004)	0.025	−0.045
	EDSS 0	—^a^	—^a^	—^a^	—^a^
	EDSS 1 or 1.5	—^b^	—^b^	—^b^	—^b^
	EDSS 2 or 2.5	−0.077	(−0.110, −0.043)	0.000	−0.080
	EDSS 3 or 3.5	−0.100	(−0.133, −0.068)	0.000	−0.106
	EDSS 4 or 4.5	−0.246	(−0.282, −0.210)	0.000	−0.265
	EDSS 5 or 5.5	−0.353	(−0.400, −0.306)	0.000	−0.279
	EDSS 6 or 6.5	−0.434	(−0.470, −0.398)	0.000	−0.673
	EDSS 7 or 7.5	−0.580	(−0.624, −0.537)	0.000	−0.718
	EDSS 8 or 8.5	−0.792	(−0.856, −0.729)	0.000	−0.451
	EDSS 9 or 9.5	—^b^	—^b^	—^b^	—^b^
	Constant	0.932	(0.900, 0.964)	0.000	—
MSIS-8D	Gender – Male	—^a^	—^a^	—^a^	—^a^
	Gender – Female	0.001	(−0.011, 0.013)	0.910	0.002
	Current Age	0.002	(0.001, 0.003)	0.000	0.129
	Time Since Diagnosis	0.000	(0.000, 0.001)	0.354	0.017
	Current MS – RRMS	—^a^	—^a^	—^a^	—^a^
	Current MS – PPMS	0.003	(−0.015, 0.021)	0.731	0.007
	Current MS – SPMS	−0.015	(−0.032, 0.002)	0.085	−0.036
	EDSS 0	—^a^	—^a^	—^a^	—^a^
	EDSS 1 or 1.5	—^b^	—^b^	—^b^	—^b^
	EDSS 2 or 2.5	−0.041	(−0.063, −0.020)	0.000	−0.073
	EDSS 3 or 3.5	−0.057	(−0.078, −0.036)	0.000	−0.101
	EDSS 4 or 4.5	−0.143	(−0.166, −0.120)	0.000	−0.258
	EDSS 5 or 5.5	−0.208	(−0.238, −0.179)	0.000	−0.277
	EDSS 6 or 6.5	−0.254	(−0.277, −0.231)	0.000	−0.661
	EDSS 7 or 7.5	−0.337	(−0.364, −0.311)	0.000	−0.701
	EDSS 8 or 8.5	−0.386	(−0.426, −0.345)	0.000	−0.369
	EDSS 9 or 9.5	—^b^	—^b^	—^b^	—^b^
	Constant	0.816	(0.796, 0.836)	0.000	—
MSIS-8D-P	Gender – male	—^a^	—^a^	—^a^	—^a^
	Gender – female	0.003	(−0.009, 0.014)	0.649	0.007
	Current Age	0.002	(0.001, 0.002)	0.000	0.128
	Time Since Diagnosis	0.000	(0.000, 0.001)	0.225	0.023
	Current MS – RRMS	—^a^	—^a^	—^a^	—^a^
	Current MS – PPMS	0.006	(−0.012, 0.023)	0.529	0.012
	Current MS – SPMS	−0.014	(−0.030, 0.003)	0.101	−0.035
	EDSS 0	—^a^	—^a^	—^a^	—^a^
	EDSS 1 or 1.5	—^b^	—^b^	—^b^	—^b^
	EDSS 2 or 2.5	−0.036	(−0.055, −0.017)	0.000	−0.067
	EDSS 3 or 3.5	−0.050	(−0.068, −0.031)	0.000	−0.092
	EDSS 4 or 4.5	−0.128	(−0.149, −0.107)	0.000	−0.241
	EDSS 5 or 5.5	−0.194	(−0.222, −0.166)	0.000	−0.269
	EDSS 6 or 6.5	−0.238	(−0.259, −0.217)	0.000	−0.646
	EDSS 7 or 7.5	−0.319	(−0.344, −0.294)	0.000	−0.691
	EDSS 8 or 8.5	−0.367	(−0.405, −0.329)	0.000	−0.366
	EDSS 9 or 9.5	—^b^	—^b^	—^b^	—^b^
	Constant	0.842	(0.824, 0.860)	0.000	—

CI, confidence interval; EDSS, Expanded Disability Status Scale; MS, multiple sclerosis; MSIS-8D, Multiple Sclerosis Impact Scale Eight Dimensions; MSIS-8D-P, Multiple Sclerosis Impact Scale Eight Dimensions Patient Version; PBM, preference-based measure; PPMS, primary progressive multiple sclerosis. RRMS, relapsing-remitting multiple sclerosis; SPMS, secondary progressive multiple sclerosis.

^a^ The reference categories were a gender of male, a current MS type of RRMS and an EDSS score of 0.

^b^ Not reported as observations available from fewer than 30 participants.

### Utility values by participant characteristics

This section describes trends in the mean utility values by participant characteristics, whilst the presence of significant relationships is explored in the regression analysis in the following section.

**Figure 1.**
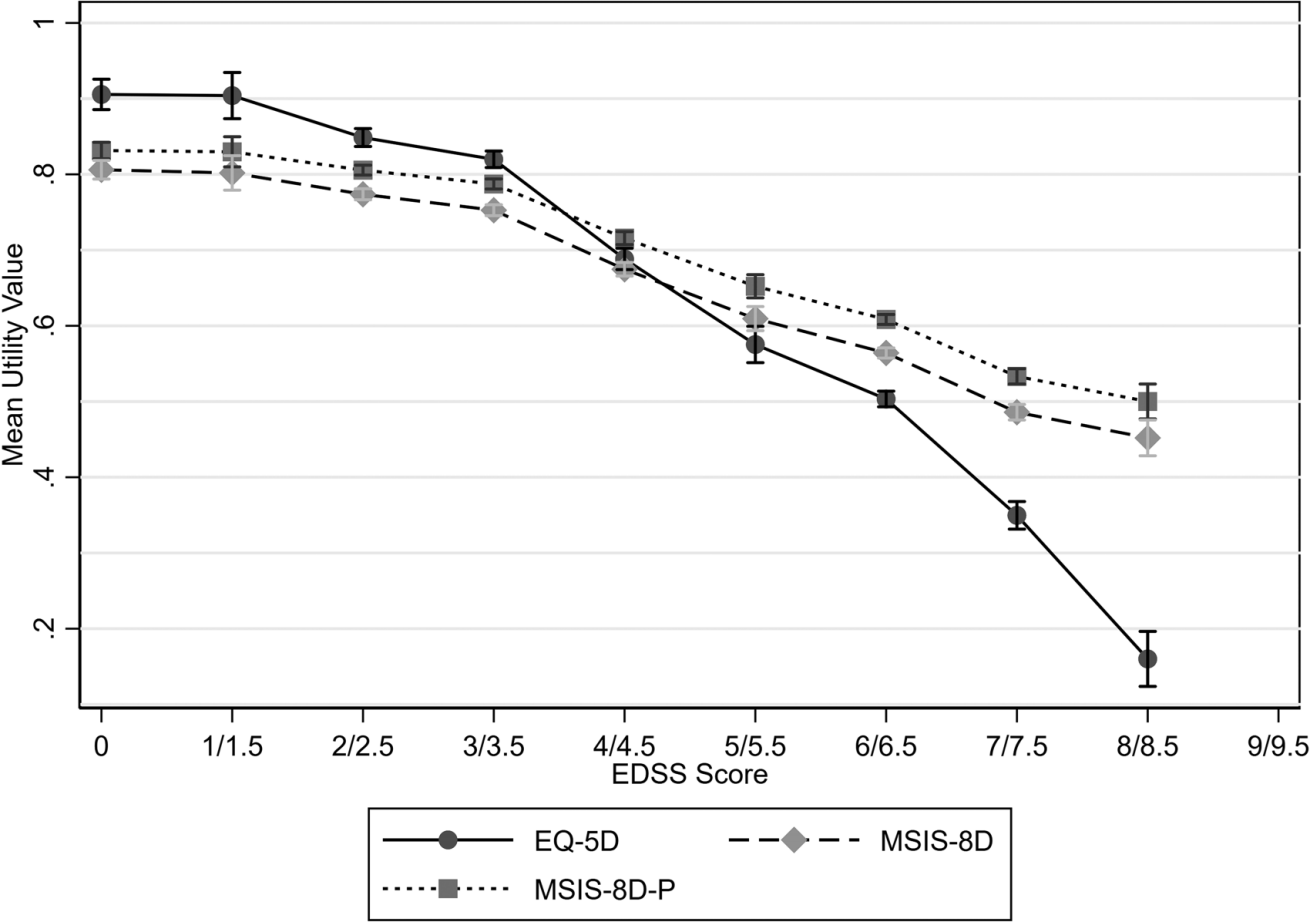
Mean utility values by EDSS score with 95% confidence intervals.

EDSS, Expanded Disability Status Scale; MSIS-8D, Multiple Sclerosis Impact Scale Eight Dimensions; MSIS-8D-P, Multiple Sclerosis Impact Scale Eight Dimensions Patient Version. Mean utility values for an EDSS score of 9 or 9.5 were not plotted as the number of participants with this score was less than 30.

Utility values decreased as EDSS scores increased, with mean EQ-5D values ranging from 0.906 for an EDSS score of 0 to 0.16 for EDSS scores of 8 or 8.5, whilst mean MSIS-8D values ranged from 0.806 to 0.452 for these EDSS scores. There was little change in utility values between EDSS scores of 0 and 1 or 1.5 (Figure 1 and Table 3).

**Figure 2.**
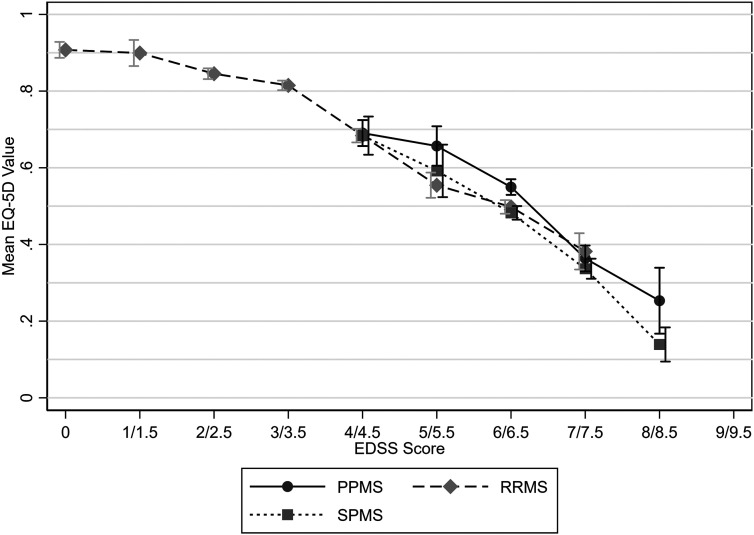
Mean EQ-5D values by EDSS score and MS type with 95% confidence intervals.

EDSS, Expanded Disability Status Scale; MS, multiple sclerosis; PPMS, primary progressive multiple sclerosis; RRMS, relapsing-remitting multiple sclerosis; SPMS, secondary progressive multiple sclerosis. Mean EQ-5D values were not plotted when the number of participants with a given EDSS score and MS type was less than 30.

**Figure 3.**
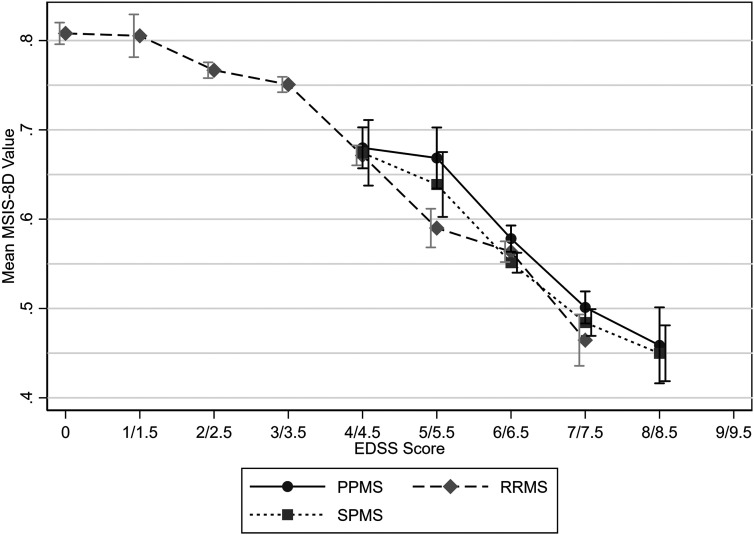
Mean MSIS-8D values by EDSS score and MS type with 95% confidence intervals.

EDSS, Expanded Disability Status Scale; MS, multiple sclerosis; MSIS-8D, Multiple Sclerosis Impact Scale Eight Dimensions; PPMS, primary progressive multiple sclerosis; RRMS, relapsing-remitting multiple sclerosis; SPMS, secondary progressive multiple sclerosis. Mean MSIS-8D values were not plotted when the number of participants with a given EDSS score and MS type was less than 30.

**Figure 4.**
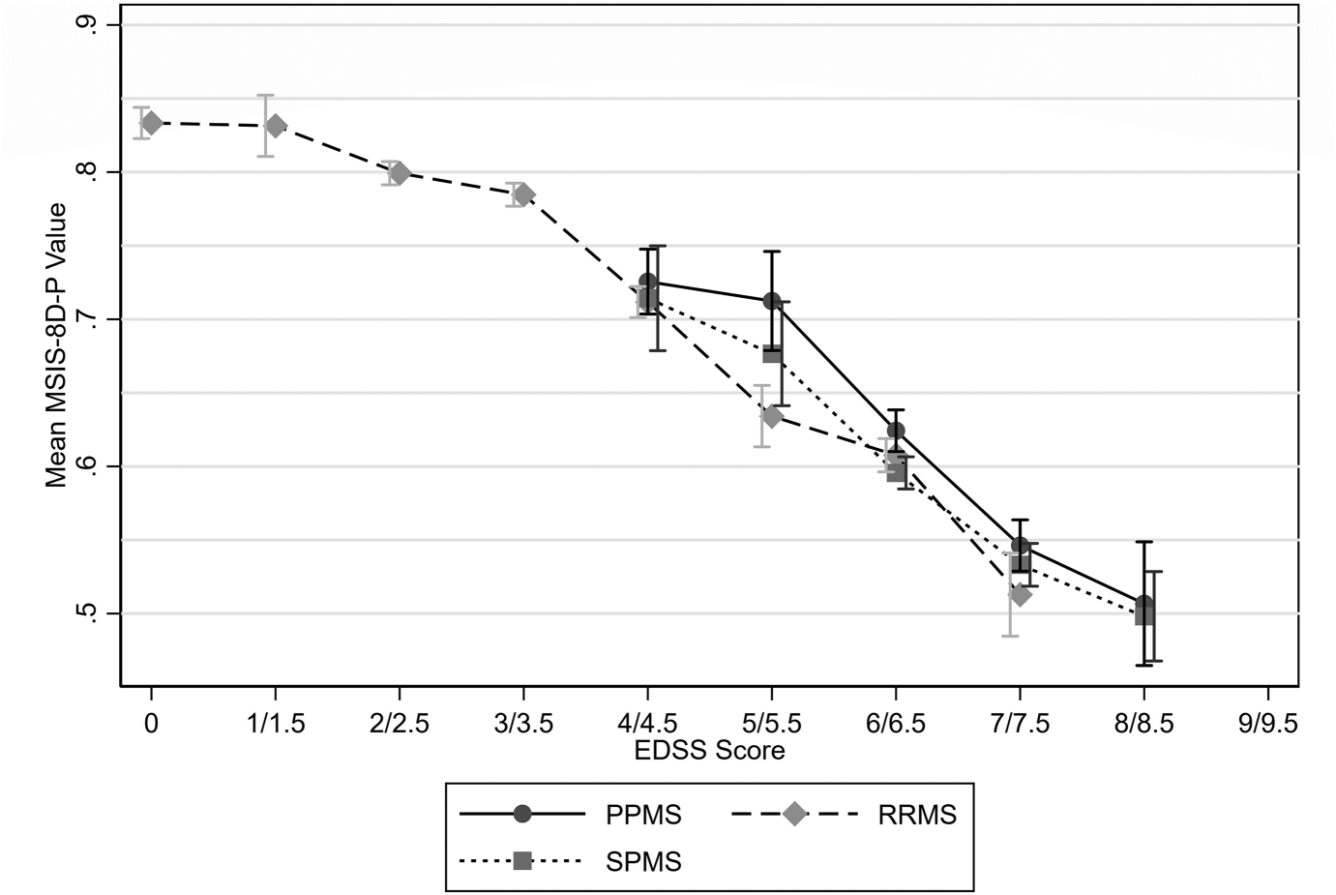
Mean MSIS-8D-P values by EDSS score and MS type with 95% confidence intervals.

EDSS, Expanded Disability Status Scale; MS, multiple sclerosis; MSIS-8D-P, Multiple Sclerosis Impact Scale Eight Dimensions Patient Version; PPMS, primary progressive multiple sclerosis; RRMS, relapsing-remitting multiple sclerosis; SPMS, secondary progressive multiple sclerosis. Mean MSIS-8D-P values were not plotted when the number of participants with a given EDSS score and MS type was less than 30.

Mean utility values were highest for relapsing-remitting MS (RRMS) and lowest for SPMS (Table 3). Similar mean utility values were observed between MS types for EDSS scores of 4 and 4.5 and 7 and 7.5. However, higher mean utility values were observed for PPMS for EDSS scores of 5 and 5.5, 6 and 6.5, and 8 and 8.5. Comparison at EDSS scores below 4 was not possible due to the small sample size of people with PPMS or SPMS at these scores, and likewise due to the lack of people with RRMS with an EDSS score of 8 or 8.5 (Figures 2–4 and Table 4).

**Figure 5.**
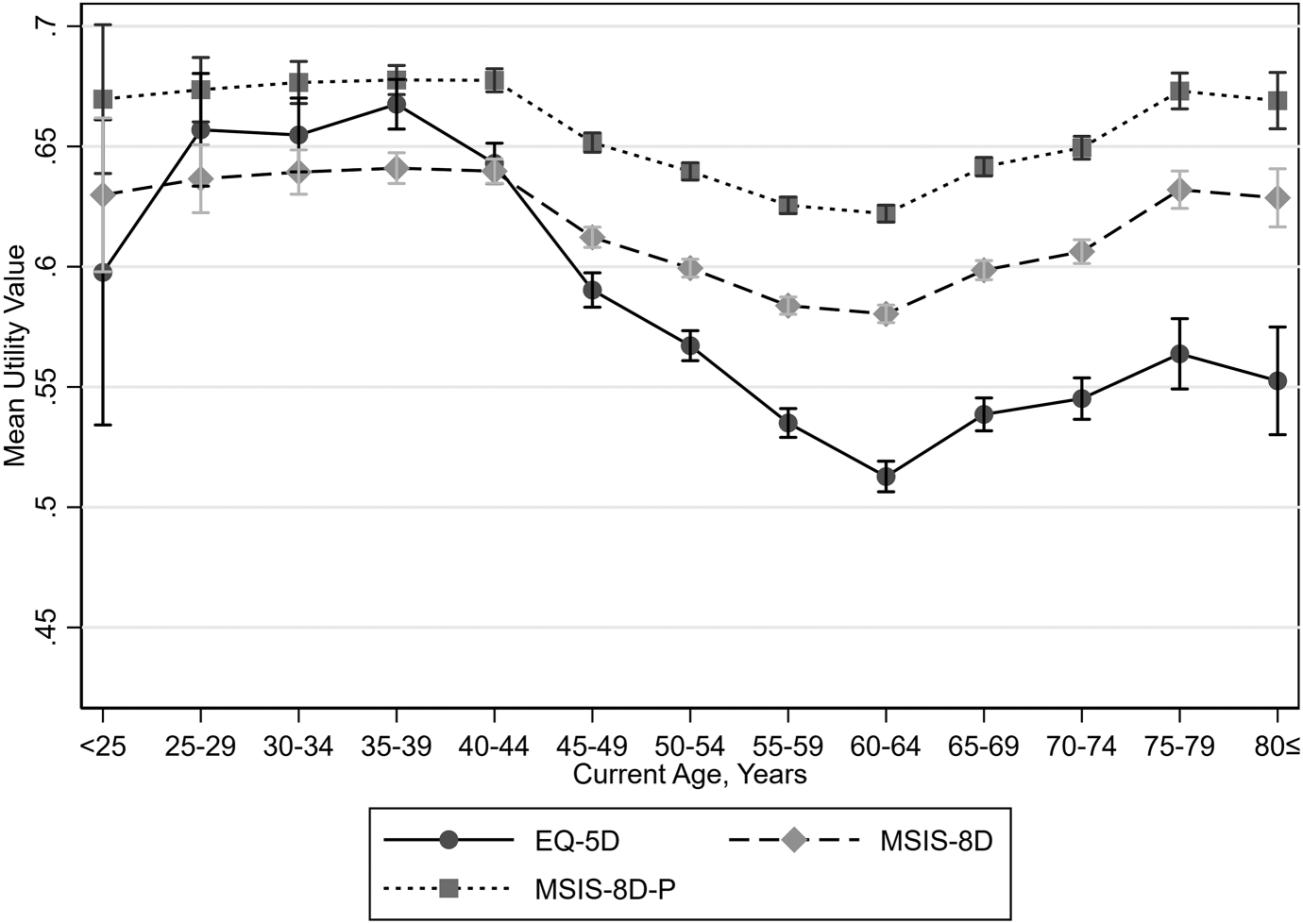
Mean utility values by current age with 95% confidence intervals.

MSIS-8D, Multiple Sclerosis Impact Scale Eight Dimensions; MSIS-8D-P, Multiple Sclerosis Impact Scale Eight Dimensions Patient Version.

Mean EQ-5D values increased from people under 25 to people aged 35 to 39, then decreased to people aged 60 to 64. Mean EQ-5D values then increased for people aged 75 to 79, with a slight drop for people aged 80 or more. MSIS-8D values demonstrated a similar pattern with current age, although smaller differences in mean values between groups were observed. There was also only a slight increase in mean MSIS-8D values from people aged under 25 to people aged 35 to 39 (Figure 5 and Table 3).

**Figure 6.**
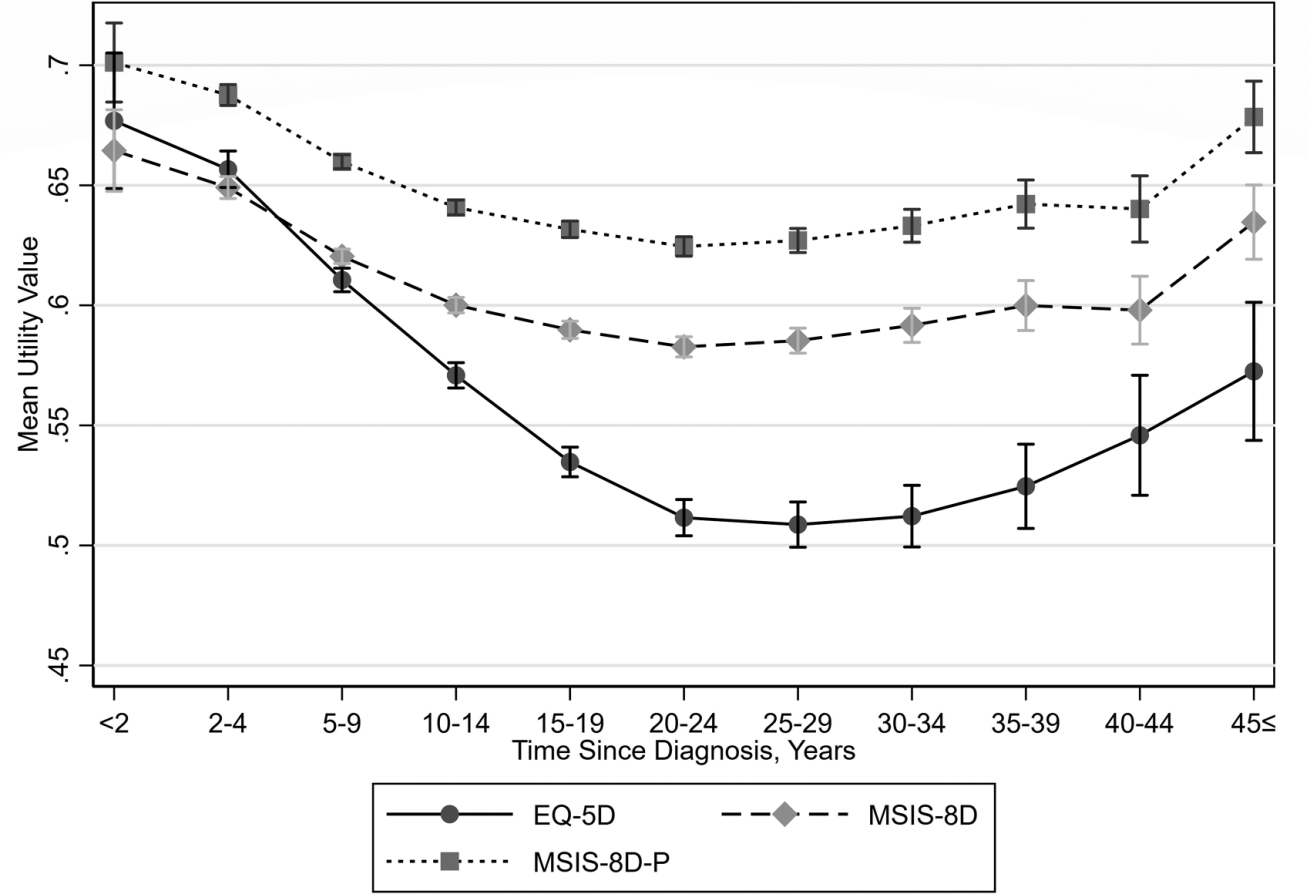
Mean utility values by time since diagnosis with 95% confidence intervals.

MSIS-8D, Multiple Sclerosis Impact Scale Eight Dimensions; MSIS-8D-P, Multiple Sclerosis Impact Scale Eight Dimensions Patient Version.

Mean utility values decreased to 20 to 24 years after diagnosis, then remained relatively unchanged to approximately 30 to 34 years after diagnosis, then increased to 45 years or more since diagnosis (Figure 6 and Table 3). Mean utility values were slightly higher for females than males (Table 3).

### Regression

**Figure 7.**
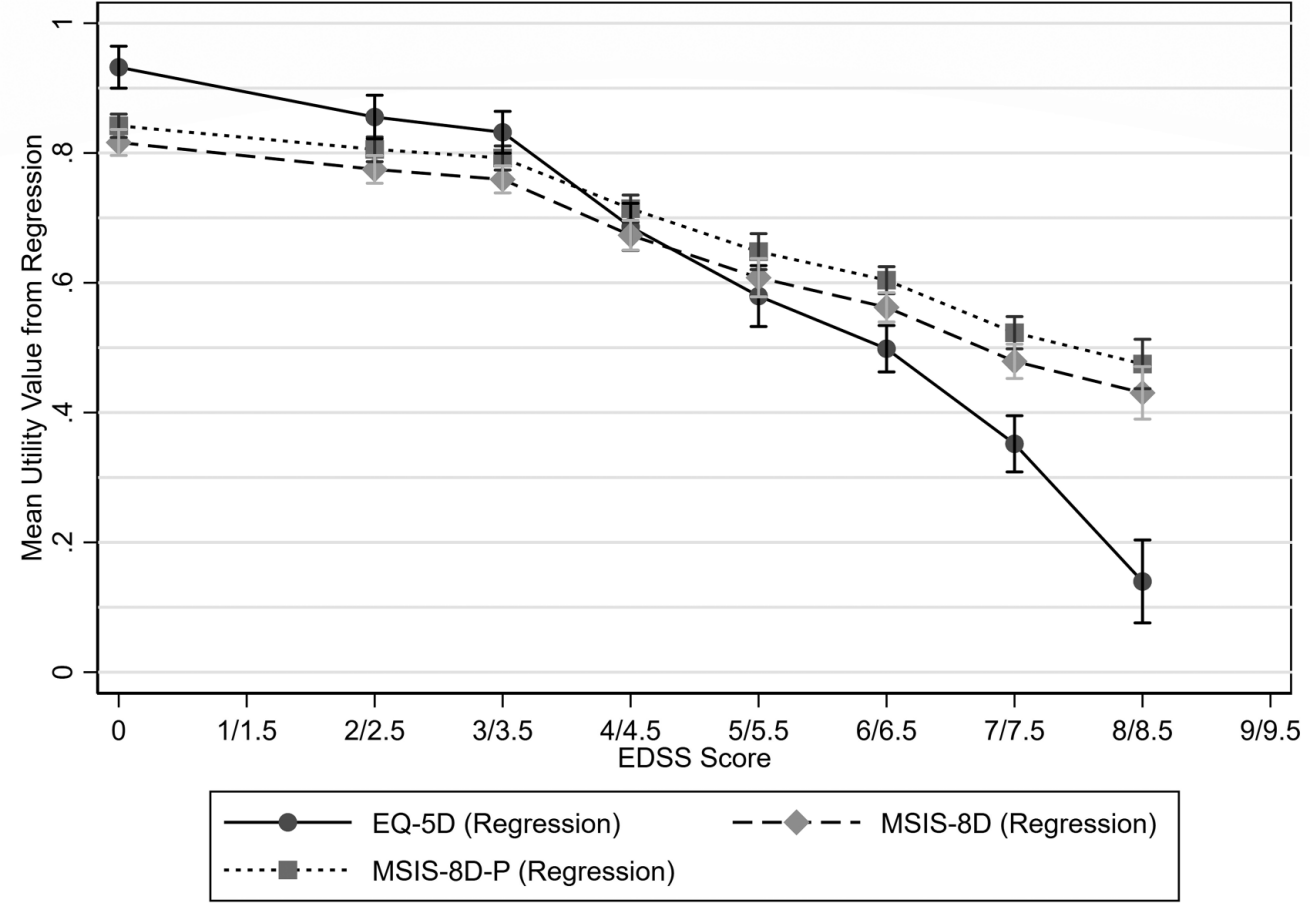
Mean utility values by EDSS score obtained from the coefficients in the robust multivariable regression with 95% confidence intervals.

EDSS, Expanded Disability Status Scale; MSIS-8D, Multiple Sclerosis Impact Scale Eight Dimensions; MSIS-8D-P, Multiple Sclerosis Impact Scale Eight Dimensions Patient Version. Results for an EDSS score of 1 or 1.5 and an EDSS score of 9 or 9.5 were not plotted as the number of participants with these scores was less than 30.

The regression analyses showed a negative association between EDSS scores and utility values (*p* < 0.001) and a positive association between age and utility values (*p* < 0.001). There was also a negative association between having SPMS and EQ-5D values (*p* = 0.025), but this was not statistically significant for the MSIS-8D (*p* = 0.085, Table 5). EQ-5D values for each EDSS score obtained from the regression coefficients were largely similar to those obtained from mean values (Figure 7).

## Discussion

This study provides valuable new information on the health-related quality of life of people with MS in the form of utility values. It utilises information from a large, representative sample of people with MS in the UK.

The EQ-5D and MSIS-8D were both found to be sensitive to differences in participant demographic and clinical characteristics. Compared to EQ-5D values, mean MSIS-8D values were often higher and demonstrated smaller absolute differences between different participant groups. This can be attributed to the smaller value range of the MSIS-8D, as compared to the EQ-5D (Table 1), which reduces the amount of possible change. This smaller range is a common characteristic of CS-PBMs, with a range of 0.4 and 0.42 to 1 for the MSIS-PBM and NQU respectively^11,16^ and 0 to 1 for the PBMSI.^15^ Use of patient tariff values also resulted in higher mean utility values and smaller absolute differences between characteristics, as observed in the literature.^23^ The smaller absolute differences observed when using a CS-PBM or patient tariff could impact the outcome of cost-effectiveness analyses.^24^ This is also observed between the EQ-5D-3L and EQ-5D-5L where the lowest possible values according to the UK tariff are −0.594 and −0.285 respectively, with critics arguing that the EQ-5D-3L may overestimate health problems and therefore underestimate utility values.^25^

**Figure 8.**
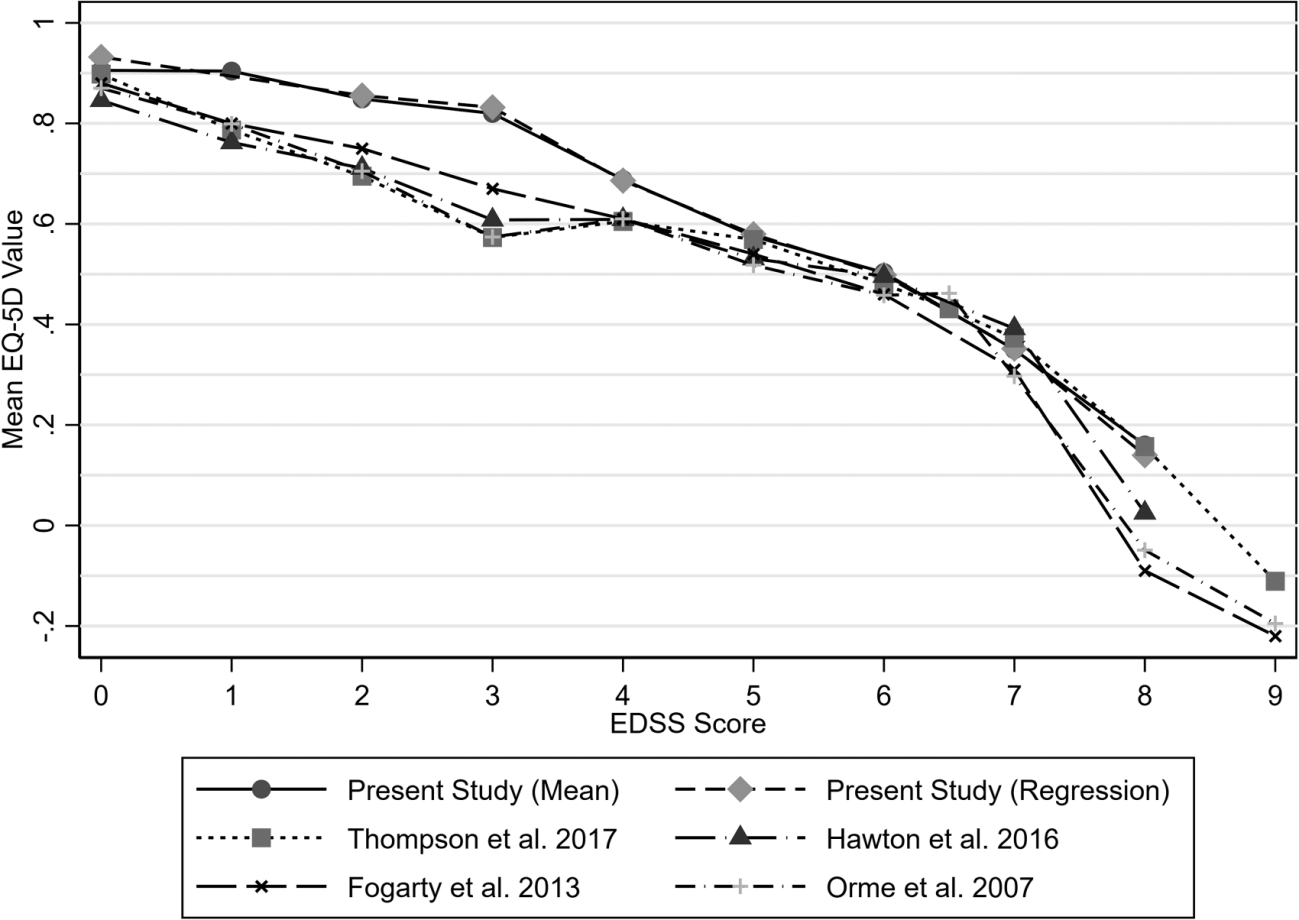
EQ-5D values by EDSS score (mean values or regression coefficients) in the present study and reported in the literature.

In some previous studies of people with MS (Figure 8), a higher mean EQ-5D value has been observed for people with an EDSS score of 4 than those with an EDSS score of 3, with increases of 0.001, 0.032 and 0.036.^4,6,9^ This inconsistency was not observed in the present study. Instead, a large decrease in the mean EQ-5D values of 0.132 was observed from EDSS scores of 3 or 3.5 to EDSS scores of 4 or 4.5. This was larger than the decrease of 0.06 observed in another relevant study of people with MS in the UK.^7^ In the present study, EQ-5D values were higher than typically observed for EDSS scores of 1 to 4.5 and 8 to 9.5. There was also little change in mean utility values between EDSS scores of 0 and 1 or 1.5, whilst other studies observed a decrement in EQ-5D values of 0.07 to 0.11.^4,6,7,9^ These differences could be due to the use of the web-EDSS in the present study. Hawton et al. 2016^4^ and Fogarty et al. 2013^7^ used clinician-rated EDSS, whilst Thompson et al. 2017^9^ used a self-report EDSS measure, although the specific measure used is not reported, and Orme et al. 2007^6^ used the self-report Adapted Patient Determined Disease Steps (APDDS).^6^ There is limited information on the comparability of the APDDS with clinician-rated EDSS. However, multiple studies have found the web-EDSS to give higher scores than the clinician-rated EDSS, with greater agreement observed for EDSS scores more than 5.^21,26,27^ This may help explain why higher average EQ-5D values were observed for some EDSS scores. It could be argued that self-report EDSS measures provide a more accurate estimation of disease severity as these are based on all of an individual's knowledge and lived experience with MS, as opposed to the restricted perspective that a clinician is likely to have.

EDSS, Expanded Disability Status Scale; MS, multiple sclerosis. EQ-5D values were obtained from studies by Thompson et al. (2007)^9^ and Hawton et al. (2016)^4 ^(mean values) and Fogarty et al. (2013)^7 ^and Orme et al. (2007)^6^ (regression coefficients). These studies were identified using a search for papers reporting EQ-5D values for people with MS in the United Kingdom using U.K. tariff values. The mean EQ-5D value for an EDSS score of 9 or more from the present study was not plotted as the number of participants with this score was less than 30. Results from the regression for an EDSS score of 1 or 1.5 and an EDSS score of 9 or 9.5 were not plotted as the number of participants with these scores in the cross-sectional sample used in the regression was less than 30.

Higher mean utility values were observed for participants with RRMS, which is consistent with the literature. Whether more severe utility values are found for PPMS or SPMS varies in the literature,^4–7^ but this study found lower mean values for SPMS than PPMS. In the regression analyses, SPMS was only significantly associated with EQ-5D values, and PPMS was not significantly associated with values on any of the PBMs. In a previous study investigating EQ-5D values by EDSS score and MS type, an increase in mean EQ-5D values between the EDSS scores of 3 to 4 and 5 to 6 was observed for people with RRMS.^4^ This was not observed in the present study, with mean EQ-5D values decreasing between each EDSS score for all MS types. In the NICE appraisal of Ofatumumab (TA699),^28^ the evidence review group's clinical expert and companies in the technical engagement responses commented that EQ-5D values for SPMS are expected to be lower than those for RRMS at the same EDSS scores.^28^ This pattern is not observed in the present study, with similar mean EQ-5D values found for people with SPMS and RRMS at each EDSS score. In cost-effectiveness analyses, it is common that RRMS is used as a base case and utility values for SPMS are calculated by adding the relevant coefficient from the regression analysis. A coefficient of −0.045 is frequently used, which is based on Orme et al. 2007,^6^ and likewise a negative coefficient of −0.031 was observed in the present study.

Women were found to only have slightly higher mean utility values than men and no statistically significant differences are found in the EQ-5D values of men versus women with MS in the UK in the literature,^6,7^ except for by Jones et al. 2013.^5^

Mean utility values decreased from less than 2 years since diagnosis to 20 to 24 years since diagnosis and from a current age of 35 to 39 years to an age of 60 to 64 years, which was anticipated. However, an increase in average utility values is then observed up to an age of 75 to 79 years and up to 45 years or more since diagnosis, with Hawton et al. 2016^4^ observing a similar improvement in utility values for time since diagnosis.^4^ This may be due to people adapting to having MS and due to differences in the reference point of older and younger individuals when making judgements on their HRQoL.^29^ In the literature, time since diagnosis has been found to have a statistically significant positive^6^ and negative^7^ association with utility values. However, in the present study, time since diagnosis was not significantly associated with utility values in the regression analysis, indicating that associations may be an artefact of other variables like age, which was significantly negatively associated with utility values, as also observed by Fogarty et al. (2013).^7^

There are limitations to this research. Some discrepancies have been observed between the web-EDSS and clinician-rated EDSS scores, meaning that scores from these measures may not be directly comparable.^21,26,27^ However, a good level of agreement has generally been demonstrated between the measures, and the recent introduction of the web-EDSS to the UK MS Register is beneficial in allowing easy, regular assessment of disease severity. The relationship between utility values and various demographic and clinical characteristics was examined, although other relevant factors like relapse rate,^4^ educational attainment, and employment status^5^ were not included. The UK MS Register study design may be open to non-response bias and volunteer effects, but the use of a prospective online cohort study enables the regular collection of real-world EQ-5D and MSIS-8D values from a large, ongoing sample. The UK MS Register does not use the EQ-5D-5L so it was not possible to investigate responses to that version of the measure.

In conclusion, this study provides utility values for a large sample of people with MS in the UK using a generic PBM and a CS-PBM using tariff values based on preferences of the general public or people with MS. An inconsistency frequently observed in previous studies, whereby average EQ-5D values increased between people with EDSS scores of 3 to 4, was not observed in the present study. Further research into whether MS disease severity is more accurately captured using self-report EDSS measures or clinician-rated EDSS would be beneficial. Overall, mean utility values were lower for SPMS than PPMS, although utility values were similar between MS types at each EDSS score, contrary to previous comments anticipating more severe values for SPMS than RRMS at the same EDSS score. However, as EDSS was measured using a self-report tool, values may not necessarily be applicable to clinician-rated EDSS scores.

This research provides usable inputs for economic evaluations. EQ-5D values can be used to calculate QALYs in cost-effectiveness analyses and the impact of using utility values from a CS-PBM and patient tariff values could also be assessed in sensitivity analyses using the average MSIS-8D and MSIS-8D-P values reported.^3^ In the last 25 years, the availability of new disease-modifying therapies has changed MS treatment strategies and resulted in an altered profile of people with MS.^30^ This study provides utility values that should better reflect current treatment practices as utility values were calculated from data collected up to 2019. Regression analyses provide insight into the unique relationship between particular demographic and clinical characteristics and utility values.
